# Effect of Thermocycling on the Bond Strength of Self-Adhesive Resin Cements Used for Luting CAD/CAM Ceramics to Human Dentin

**DOI:** 10.3390/ijms23020745

**Published:** 2022-01-11

**Authors:** Andrzej Malysa, Joanna Wezgowiec, Wojciech Grzebieluch, Dariusz P. Danel, Mieszko Wieckiewicz

**Affiliations:** 1 Department of Experimental Dentistry, Wroclaw Medical University, 50-425 Wroclaw, Poland; andrzej.malysa@umw.edu.pl (A.M.); joanna.wezgowiec@umw.edu.pl (J.W.); 2 Department of Conservative Dentistry, Wroclaw Medical University, 50-425 Wroclaw, Poland; wojciech.grzebieluch@umw.edu.pl; 3 Department of Anthropology, Ludwik Hirszfeld Institute of Immunology and Experimental Therapy, Polish Academy of Sciences, 53-114 Wroclaw, Poland; dariusz.danel@gmail.com

**Keywords:** shear bond strength, self-adhesive resin cement, adhesion, luting agents, tooth, dentin, CAD/CAM ceramic, thermocycling, artificial aging

## Abstract

The aim of the study was to evaluate the influence of thermocycling on the shear bond strength of self-adhesive, self-etching resin cements luted to human dentin and computer-aided design/computer-aided manufacturing (CAD/CAM) ceramics. Three modern self-adhesive dental cements (Maxcem Elite, RelyX U200, Panavia SA) were used to lute three CAD/CAM ceramics (IPS Empress CAD, IPS e.max CAD, IPS e.max ZirCAD) onto the dentin. One conventional cement (Panavia V5) served as a control. After preparation, the samples were subjected to thermocycling as a method of artificial aging of dental materials applied to simulate long-term use in oral conditions. Shear bond strength was evaluated according to PN-EN ISO 29022:2013-10 and failure modes were observed under a light microscope. Statistical analysis was performed. The study demonstrated that a combination of ceramics and cements directly impacts the bond strength. The highest bond strength was observed in Panavia V5, lower in Panavia SA and Maxcem Elite and the lowest–in RelyX U200. Adhesive failure between human dentin and cements was the most common failure mode. Moreover, thermocycling highly decreased bond strength of self-adhesive, self-etching cements.

## 1. Introduction

The use of all-ceramic restorations in modern dental prosthetics is still increasing due to the development of innovative ceramic materials and computer-aided design/computer-aided manufacturing (CAD/CAM) technologies. One of the most important clinical aspects of prosthetic ceramic reconstruction is providing a suitable bond strength between ceramic surface and tooth tissues [1,2,3]. The strong adhesion achieved due to the application of proper cements directly translates into the longevity of the prosthetic restoration, especially in the case of minimally invasive restorations such as veneers, inlays or overlays [4]. In particular, stable cementation of ceramic materials strengthens the marginal adaptation, protects against micro-leakage and significantly reduces the risks of cracking and fracturing of the restoration [5,6]. As a result, it is crucial for functional properties of the restoration, influencing both its mechanical and biological behavior.

The choice of cement is one of the most important steps to obtain a strong bond between dental ceramics and the hard tissues of the teeth [3,4,5,6]. The use of conventional resin-based cements with well-developed methods of surface modification allows us to obtain a high-quality bond of ceramic restoration to a tooth [7,8,9,10]. However, due to the multitude of surface modification techniques, duration and complexity of the procedure, as well as the dependence on the operator skills, simplification of the methodology has become necessary to reduce the risk of error. For this reason, contemporary technology, in order to meet the growing expectations, has developed self-etching, self-adhesive cements.

Using 10-methacryloyloxydecyl dihydrogen phosphate (MDP) or long carbon-chain silane (LCSi) coupling agent monomers in modern self-adhesive, self-etch cements is claimed to ensure high durability of the joined surfaces and elimination of additional steps needed to obtain proper bond strength of the prosthetic reconstruction to the tooth tissues [11,12]. On the other hand, research shows large discrepancies in the bond strengths of modern self-adhesive cements based on MDP monomers, particularly in the cases when they are used for cementation of zirconium materials [8,9,10,11,12,13]. The selection of an appropriate cementing protocol or modifications of ceramic surface directly affects the obtained bond strength results [14,15,16].

Our previous research evaluated the potential of self-adhesive, self-etching cements for the cementation of the selected CAD/CAM ceramic materials from various groups (glass ceramics, lithium disilicate and zirconium) [17]. The obtained shear forces depended on the selection of cement in relation to the cemented ceramic material. The study revealed much a higher bond strength of conventional cement than self-adhesive, self-etching cements. To obtain a broader view concerning long-term behavior, the bond strength analysis of dental materials should also apply some type of accelerated aging method [14,15,16,17,18,19,20].

The artificial decrease of bond strength over time better reflects the real behavior and characteristics of the cement and, therefore, is necessary during in vitro studies. However, a single optimal method of accelerated aging perfectly mimicking intraoral conditions has not yet been developed. Various approaches and protocols have been described, based on thermocycling, thermomechanical aging, dynamic load or water storage [18,19,20,21,22,23]. In the current study, we applied thermocycling as one of the most common methods of artificial aging of dental materials [5,14]. Most of the research follows the temperature range of 5–55 °C defined in ISO/TS 11405, but they differ in terms of dwell time and number of cycles performed [5,6,14,16].

The present study aimed to assess the shear bond strength of self-adhesive, self-etching resin cements used for luting CAD/CAM ceramics to human dentine when then samples were subjected to artificial aging (thermocycling). The following research hypothesis was tested: there is no significant difference between the shear bond strength of the modern self-adhesive resin cements and the conventional luting agent (Panavia V5). The second hypothesis was formulated as follows: there is no significant difference between the shear bond strength obtained for thermocycled and non-thermocycled samples.

## 2. Results

### 2.1. Shear Bond Strength between Ceramic and Dentin

The four types of dental cement differed (regardless of the ceramics type) in the shear bond strength (F(3, 44) = 5763.53, *p* < 0.0001, η^2^ = 0.997). The highest shear bond strength was observed in Panavia V5 (M = 14.63); this was lower in Panavia SA (M = 2.91) and RelyX U200 (M = 2.82) and the lowest in Maxcem (M = 2.58). The post-hoc Tukey’s HSD test showed statistically significant differences between Panavia V5 and all other cements (all *p*’s < 0.0002), as well as between Panavia SA and Maxcem (*p* = 0.024). The differences between RelyX U200 and Panavia SA (*p* = 0.83) and Maxcem (*p* = 0.17) were not statistically significant.

Regardless of the dental cements used, the examined ceramics differed in their shear bond strengths (F(2, 88) = 467.81, *p* < 0.0001, η^2^ = 0.91). The highest shear bond strength was observed in IPS e.max CAD (M = 6.96); this was lower in IPS Empress CAD (M = 6.64) and the lowest in IPS e.max ZirCAD (M = 3.61). The Tukey’s post-hoc tests showed that all differences between ceramics were statistically significant (IPS Empress CAD vs. IPS e.max ZirCAD: *p* = 0.0001; IPS Empress CAD vs. IPS e.max CAD: *p* = 0.02; IPS e.max ZirCAD vs. IPS e.max CAD: *p* = 0.0001).

**Figure 1:**
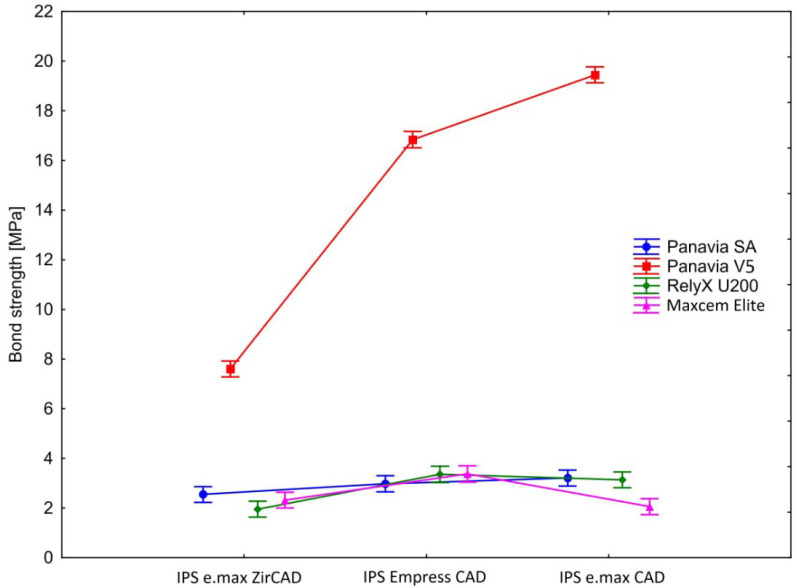
Shear bond strength in the examined samples. Vertical bars denote 0.95 confidence intervals.

The statistically significant effect of the interaction between ceramics and cement (F(6, 88) = 297.84, *p* < 0.0001, η^2^ = 0.95) indicated that the shear bond strength depends on the combination of both materials (Figure 1 and Table A1). The shear bond strength for Panavia V5 and Panavia SA increased from IPS e.max ZirCAD through IPS Empress CAD to IPS e.max CAD. For RelyX U200, the shear bond strength increased from IPS e.max ZirCAD through IPS e.max CAD to IPS Empress CAD. In the case of Maxcem, that lowest shear bond strength was observed of IPS e.max CAD, higher for IPS e.max ZirCAD and the highest for IPS Empress CAD. The post-hoc analysis by Tukey’s HSD test showed that 20 (out of 66) pairwise comparisons did not reach the statistical significance level (all *p*’s ≥ 0.14). The differences in shear bond strength between all other groups were statistically significant (all *p*’s ≤ 0.016). Detailed results of the post-hoc analysis are presented in Table A2.

In order to directly test our research hypothesis, we ran a series of planned contrast analysis (detailed results are presented in Table 1 and Figure 1). Firstly, we focused on IPS e.max ZirCAD samples and compared shear bond strength in Panavia V5 to Panavia SA, RelyX U200 and Maxcem, respectively. All planned contrasts were statistically significant, indicating that, when compared to Panavia V5, other examined types of cement were characterized by significantly lower shear bond strength (in descending order: Panavia SA, Maxcem, RelyX U200). Analogous analysis for IPS Empress CAD samples also showed that all planned contrasts were statistically significant. In this case, the Panavia V5 was characterized by the highest shear bond strength and statistically significantly different than other types of cement (in descending order: Maxcem, RelyX U200, Panavia SA). In the last series of planned contrasts, we concentrated on the IPS e.max CAD samples and compared the shear bond strength in Panavia V5 to other types of cement. As in the previous cases, all planned contras were statistically significant. Comparing to Panavia V5 all other types of cement were characterized by significantly lower shear bond strength (in descending order: Panavia SA, RelyX U200, Maxcem).

### 2.2. Microscopic Evaluation of a Failure Mode

**Figure 2:**
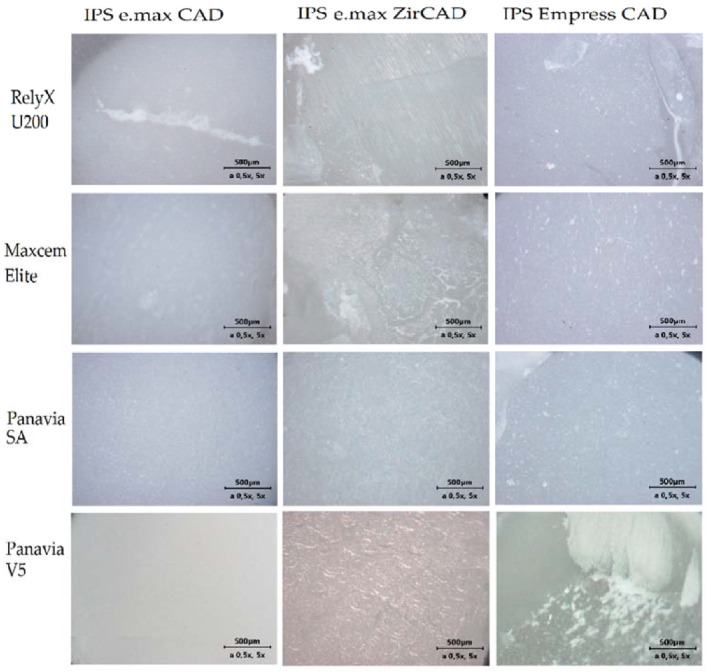
Light microscope photographs of different failure modes observed on ceramic surfaces.

**Figure 3:**
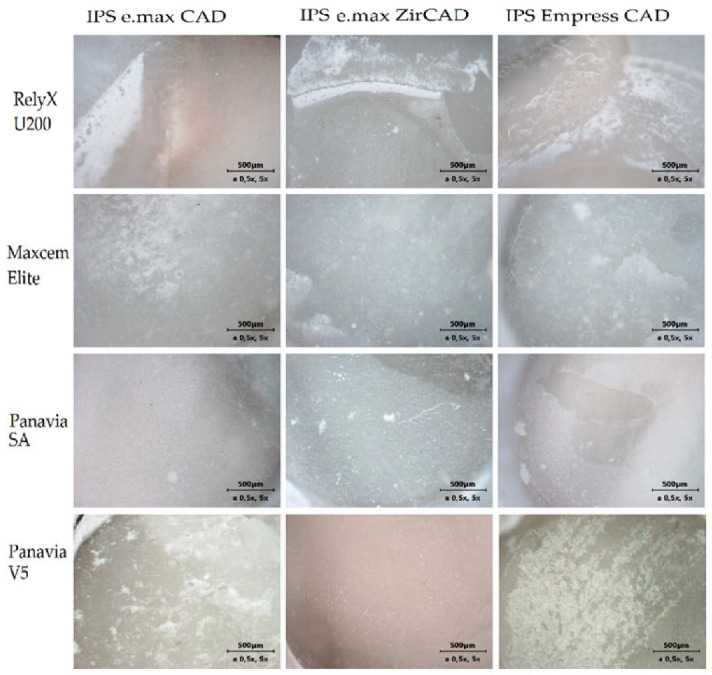
Light microscope photographs of different failure modes observed on dentin surfaces.

The microscopic photographs of the sheared samples surfaces are presented in Figure 2 (surfaces of ceramic cylinders) and in Figure 3 (surfaces of human dentin). Quantitative results of the failure mode analysis were summarized in Table 2. It was found that the adhesive failure between dentin and cement were predominant mode among the self-etching, self-adhesive cements with the selected ceramics. Application of Panavia V5 cement in case of IPS Empress CAD ceramics most often resulted in a mixed failure. However, in the case of IPS e.max ZirCAD and IPS e.max CAD, an adhesive failure at cement/dentin interface was a predominant mode, similarly to the studied self-adhesive, self-etching cements (Table 2).

### 2.3. Comparison of Shear Bond Strength for Thermocycled and Non-Thermocycled Samples

The results of the current study were compared with the results of the previous research carried out using exactly the same materials and methodology, but without thermal accelerated aging [17].

**Figure 4:**
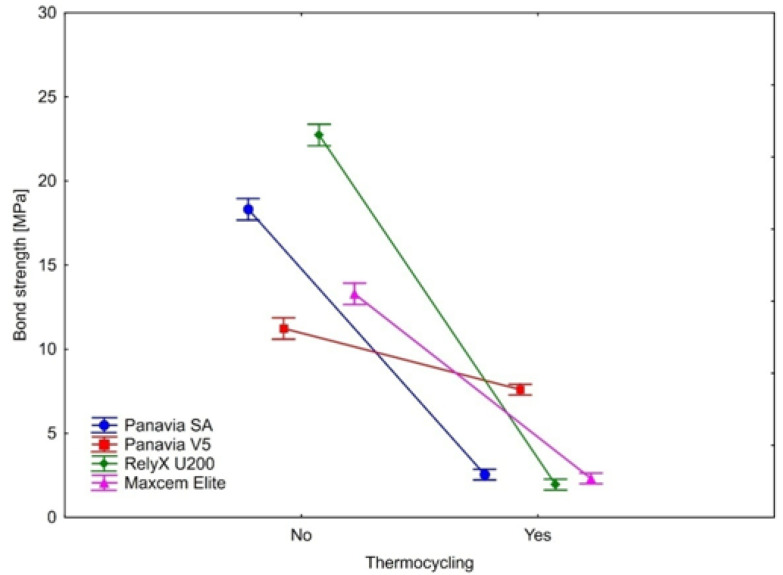
The effect of thermocycling on luting cement bond strength to IPS e.max ZirCAD. Vertical bars denote 0.95 confidence intervals around the mean values.

**Figure 5:**
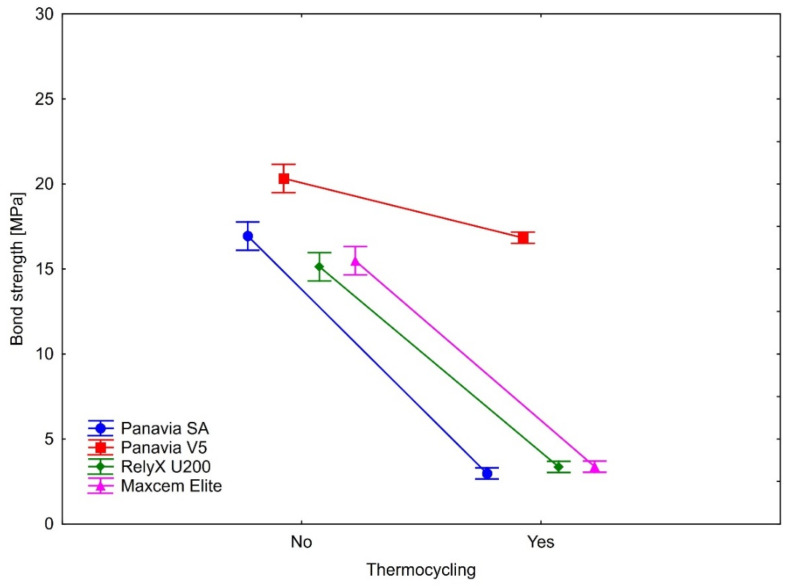
The effect of thermocycling on luting cements bond strength to IPS Empress CAD. Vertical bars denote 0.95 confidence intervals around the mean values.

**Figure 6:**
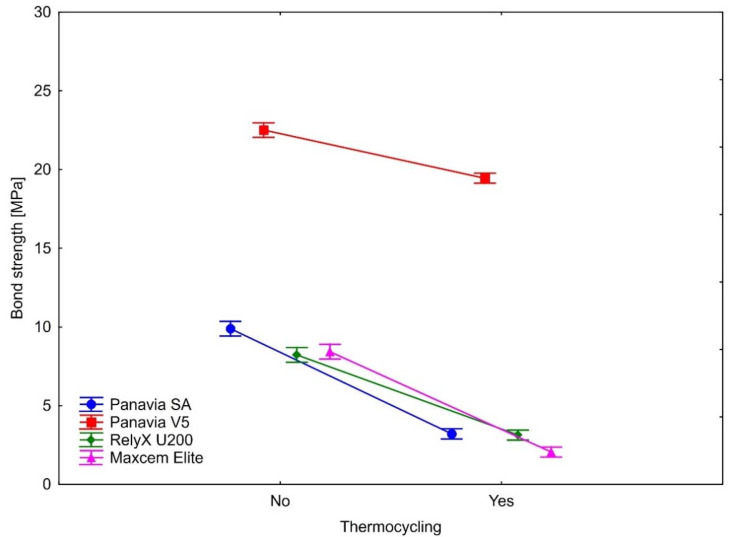
The effect of thermocycling on luting cements bond strength to IPS e.max CAD. Vertical bars denote 0.95 confidence intervals around the mean values.

The bond strength after thermocycling was statistically significantly weaker in all samples. This observation applies to all four types of cement on three ceramics. As shown by the calculated effect sizes, the magnitudes of the observed differences in all cases were substantial, indicating the large effect of aging (thermocycling) on the bond strengths. Detailed results are presented in Table 3 and Figure 4, Figure 5 and Figure 6.

Complimentary analyses showed that thermocycling affected the magnitude of changes in the bond strength differently depending on the type of cement. This effect was statistically significant for all three ceramics (IPS e.max ZirCAD: F(3, 44) = 449.387, *p* < 0.0001, η^2^ = 0.97); IPS Empress CAD: F(3, 44) = 104.358, *p* < 0.0001, η^2^ = 0.88); IPS e.max CAD: F(3, 44) = 35.795, *p* < 0.0001, η^2^ = 0.71).

In the case of IPS e.max ZirCAD, the lowest decline in the bond strength was observed for Panavia V5, larger in Maxcem and Panavia SA. The largest drop in the bond strength was observed for RelyX U200 (Table 3 and Figure 4). Tukey’s post-hoc tests showed that the differences in the bond strength decline were statistically significant between all cement types (all *p*’s = 0.0002).

Similarly, for the IPS Empress CAD, Panavia V5 had the lowest decline in the bond strength (Table 3 and Figure 5). Larger decline values were observed (in ascending order) for RelyX U200, Maxcem and Panavia SA. While the RelyX U200 and Maxcem were not statistically significantly different in the magnitude of bond strength decline (*p* = 0.95), the other comparisons showed that the magnitudes of change in the bond strength were statistically significant between analyzed types of cement (Panavia SA vs. Panavia V5: *p* = 0.0002; Panavia SA vs. RelyX U200: *p* = 0.008; Panavia SA vs. Maxcem: *p* = 0.03; Panavia V5 vs. RelyX U200: *p* = 0.0002; Panavia V5 vs. Maxcem: *p* = 0.0002).

In the case of IPS e.max CAD, the pattern of changes in the bond strength were analogous as in IPS Empress CAD. Again, the lowest decline was observed for Panavia V5. Larger values were observed (in ascending order) for RelyX U200, Maxcem and Panavia SA (Table 3 and Figure 6). The magnitude of the bond strength declines did not statistically significantly differ between Panavia SA and Maxcem (*p* = 0.86). Statistically significant differences were observed between other types of cement (Panavia SA vs. Panavia V5: *p* = 0.0002; Panavia SA vs. RelyX U200: *p* = 0.001; Panavia V5 vs. RelyX U200: *p* = 0.0002; Panavia V5 vs. Maxcem: *p* = 0.0002; RelyX U200 vs. Maxcem: *p* = 0.01).

## 3. Discussion

The essence of the clinical long-term success of all-ceramic prosthetic restoration is not only related to the mechanical strength of ceramic, but also to the durable and strong adhesion of ceramics material to the tooth’s hard tissues provided by the proper cementation. A strong need for a reliability and resistance to the oral cavity environment is a particularly crucial aspect of minimally invasive reconstructions [3,7,8,9]. Especially the durability of veneers, overlays, inlays, etc., directly depends on the surface adhesion [7]. Durable ceramic-tooth tissue connection relies on chemical bonding and micromechanical interlocking [12,15].

Nowadays, the subject of adhesion of modern high-strength ceramics, and also those processed in digital CAD/CAM technologies, is intensively researched in many aspects. A large number of studies focuses on the modification of the ceramic surface in order to obtain the highest possible bond strength [1,2,3,4,5,6,7,8,12,13,14,15]. In opposition to this point of view is the approach emphasizing the potential of the use of self-adhesive self-etching cements, which significantly reduces the number of steps involved in preparation of the surfaces to be joined and, as a result, also reduces possible operator errors [19]. Despite these obvious advantages, the possible risks associated with the use of self-adhesive cements still require investigation, particularly in terms of its effect on the long-term durability of restoration.

To simulate the wear of dental materials over time, artificial aging should be performed and its effect must be assessed, e.g., during the evaluation of the strength of adhesive materials applied between tooth tissue and ceramics. These challenges remain since there is no unified protocol for simulating accelerated aging. The literature describes dynamic simulations [23,25,26] or the storage of samples in water baths at constant temperature [6,14,26]. Thermocycling is one of the most common methods to age the material; however, its parameters have not yet been unified. Comino-Garayoa et al. conducted a systematic review analyzing 45 different papers, concluding that artificial aging should be based on 5000 thermal cycles or 30 days of continuous water bath [26]. Other systematic reviews [5,9,10,26] comparing parameters of thermocycling enable us to draw the general conclusion that a gradient of temperatures from 5 °C to 55 °C should be applied. These temperatures are also in accordance with the ISO TS 11405 Technical Specification for testing the adhesion to tooth structure. On the other hand, there are still significant differences in the number of cycles performed, suggesting that this is a prediction-based parameter [10,22,23].

Our study revealed that thermocycling significantly reduced a bond strength of dental cements. The performed statistical analysis showed that the bond strengths of self-etching, self-adhesive cements were strongly dependent on the use of this method of artificial aging (or not). Noticeable, though much smaller, decrease of bond strength was also demonstrated for the conventional Panavia V5 cement used as a control group.

Results of different preclinical and clinical studies concerning self-adhesive, self-etch cements used as a luting agent for prosthetic restorations revealed that there is no doubt that conventional resin cement with proper surface modification provides better dentin bonding and sealing performance [22]. However, not only focusing on the stabilization of the restoration, but also effective and durable bonding to dentine is still a fundamental prerequisite for clinical success when using self-adhesive resin cements for the luting of ceramic restorations [21]. Some in vitro studies proved that bonding with self-adhesive, self-etch cements might be beneficial for the tooth tissue, as they may be less toxic than conventional agents [21]. On the other hand, this is in contrast to the results of Sawada et al., in which no significant differences were observed between the self-adhesive, self-etch bonding agents [13,27].

As the methods of research sample preparation and their bond strength evaluation presented in the literature differ significantly, it is impossible to conduct a reliable meta-analysis [27,28,29,30,31]. The methodology described in this study is standardized based on ISO guidelines, which provide an opportunity for their reproduction. However, the main limitation is connected with the simple method of artificial aging applied. Further research is required, taking other methods into account. Generally, it must be taken into account that resin-based luting cements, similarly as other materials used for the reconstruction of teeth hard tissues, must withstand a number of factors directly and indirectly affecting the strength and stability of the connection. It should be noted that the oral cavity environment is not only affected by temperature variations. Changes in humidity, pH, saliva enzymes effects or physical forces acting in three axes also significantly influence the maintenance of the reconstruction [19]. Shahin et al. used accelerated aging based on combined thermocycling and the dynamic loading of samples material, which seems to better reflect the oral cavity conditions [16]. Following this direction would be a valuable continuation for our current research.

## 4. Materials and Methods

The current study strictly followed a methodology applied in the previous research [17]. In this section, only a brief description is presented.

### 4.1. Samples Prepration

Detailed description of the materials used in this study is included in Table 4. Four resin cements were used: Panavia SA, RelyX U200, Maxcem Elite (self-etching, self-adhesive cements) and Panavia V5 (a conventional resin cement used as a control). Three types of ceramics were selected: IPS Empress CAD, IPS e.max CAD, IPS e.max ZirCAD. For each combination of cement and ceramics 12 samples were prepared. Following the PN-EN ISO 29022: 2013-10 standard, 144 ceramic cylinders with a diameter of 2.38 mm and a height of 5 mm were designed and milled in CAD/CAM technology using the Sirona Cerec inLAB SW 19.0 system (Sirona, New York, NY, USA). The cylinders were cemented into human dentin slices obtained from 67 freshly extracted, caries-free human molars (approved by Wroclaw Medical University Bioethical Committee, No. KB-37/2018). For this purpose, a PetroThin Thin Sectioning System with diamond disc and water cooling (Buehler, Lake Bluff, IL, USA) was used to cut the coronal dentin into 3 mm thick slices. Before cementing the ceramic cylinder, each prepared dentin specimen was grounded with a carborundum paper of P 400 granularity (Luna, Bern, Switzerland).

The cementation of ceramic cylinders onto human dentine was carried out in accordance with the manufacturer’s guidelines. For self-etching, self-adhesive cements, only the modification of the ceramic surface was performed. The IPS Empress CAD and IPS e.max CAD ceramics were etched with 9% hydrofluoric acid (3M ESPE, Maplewood, MN, USA) for 1 min. The surface of the IPS e.max ZirCAD ceramic was pretreated using CoJet System (3M ESPE, Maplewood, MN, USA). Conventional Panavia V5 cement required additional modification of dentin surfaces with 37% orthophosphoric acid (3M ESPE, Maplewood, MN, USA). Cementation of ceramics onto dentin was carried out with a fixed compression force of 10 N under the control of the FB(C) dynamometer (Axis, Gdansk, Poland). Polymerization was performed using Elipar LED lamp (3M ESPE, Maplewood, MN, USA) for 20 s. Before performing simulated artificial aging, the prepared samples were stored in distilled water at 37 °C for 24 h.

### 4.2. Artificial Aging

The process of artificial aging of the samples was performed using THE-1100 thermocycler (SD Mechatronik, Munich, Germany). Each specimen was subjected to 2000 cycles in temperatures between 5 °C and 55 °C with a dwell time of 40 s and transfer time of 15 s. Immediately after completion of accelerated aging, the samples were sheared.

### 4.3. Evaluation of the Shear Bond Strength between Ceramic and Dentin

**Figure 7:**
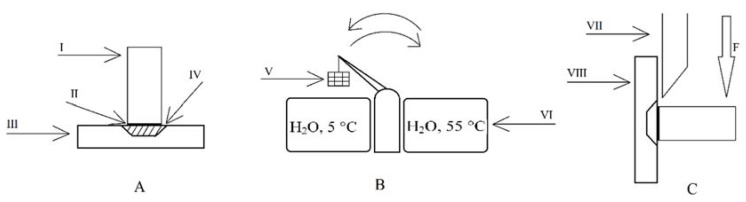
Schematic illustration of the sample preparation, thermocycling and shearing: (**A**) ceramic cylinder cemented onto human dentin embedded in an acrylic retainer; (**B**) thermocycling; (**C**) shear bond strength measurement; I-ceramic cylinder; II-resin luting cement. III and VIII-acrylic dentin slice retainer; IV-slice of human dentine; V-basket with thermocycled samples; VI-distilled water baths; VII-shear knife of the universal testing machine.

The shear bond strength tests were carried out using a universal testing machine (Thumler, Nurnberg, Germany), following PN-EN ISO 29022:2013-10 standard, with the crosshead speed of a 1-mm/min and a maximum force of 3000 N. Preparation of samples, thermocycling and shear bond strength test were presented in Figure 7.

### 4.4. Microscopic Evaluation of a Failure Mode

Failure modes were observed under light microscope (Axio Lab. A1 MAT, Zeiss, Oberkochen, Germany) with ×5 magnification. For each sample, the type of fracture was specified as a failure of: adhesion between ceramic and cement, adhesion between dentin and cement, cohesion in cement, cohesion in ceramic, cohesion in dentin or mixed failure.

### 4.5. Statistical Analysis

Mixed-design analysis of variance (split-plot ANOVA) with ceramics (i.e., IPS e.max ZirCAD, IPS Empress CAD, IPS e.max CAD) as a within-group repeated measure and dental cement (i.e., Panavia SA, Panavia V5, RalyX U200, Maxcem) as a between-group factor was used for this study. We employed a post-hoc analysis with Tukey’s honestly significant difference (HSD) test for equal sample sizes to investigate differences between all compared groups. Additionally, to directly test our predictions, we performed a planned contrast analysis.

In a second step, we used a paired sample *t*-test to compare the bond strength of four types of cement (Panavia SA, Panavia V5, RelyX U200, Maxcem) tested in two experimental conditions (with or without thermocycling). The analyses were conducted separately for three ceramics (IPS e.max ZirCAD, IPS Empress CAD, IPS e.max CAD). To estimate the effect size of the observed differences, we calculated Cohen’s d_z_ for correlated samples [24]. A one-way ANOVA was conducted to test if the changes of the bond strength resulting from thermocycling (dependent variable) are statistically different in four types of cement (independent variable). To test this effect in more detail and identify which types of cement differ in the bond strength change from each other, we ran post-hoc pairwise comparisons using Tukey’s HSD test. The complementary analyses were also conducted separately for each of the three ceramics.

A probability value of *p* < 0.05 indicated statistically significant results. All statistical analyses were conducted in Statistica (data analysis software system), version 10 (StatSoft Inc., Tulsa, OK, USA), and using Psychometrica online tools (Psychometrica—https://www.psychometrica.de/effect_size.html (accessed on 16 December 2021), Alexandra Lenhard, Dettelbach, Germany) for calculating the effect sizes for the planned contras analysis [24].

## 5. Conclusions

Within the limitations of this in vitro study, the following conclusions were drawn:

1. Conventional resin cement (Panavia V5) showed significantly higher bonding strengths compared to self-adhesive, self-etching cements after accelerated thermal aging.
2. Regardless of the tested cement, the lowest bond strength among the tested ceramics was obtained for IPS e.max ZirCAD.
3. The appropriate selection of cement for ceramics is crucial, since differences in bond strengths for the studied combinations were statistically significant.
4. Comparing samples not subjected to thermocycling to the ones that were artificially aged, the greatest decreases in bond strength were observed for self-etching self-adhesive cements.

## Figures and Tables

**Table 1:** Planned contrast analysis for differences in the shear bond strength between Panavia V5 and Panavia SA, RelyX U200, Maxcem respectively. The Cohen’s *d* for groups with equal size was calculated according to Lenhard, W. & Lenhard, A [24].

	IPS e.max ZirCAD			IPS Empress CAD			IPS e.max CAD		
Panavia V5	*t* (44)	*p*	Cohen’s *d*	*t* (44)	*p*	Cohen’s *d*	*t* (44)	*p*	Cohen’s *d*
Panavia SA	22.61	<0.0001	7.542	60.07	<0.0001	18.221	72.13	<0.0001	28.188
RelyX U200	25.26	<0.0001	8.638	58.41	<0.0001	21.586	72.46	<0.0001	28.135
Maxcem	23.63	<0.0001	9.496	58.35	<0.0001	21.783	77.25	<0.0001	34.233

**Table 2:** Quantitative results of failure mode analysis.

Ceramic	Resin Cement	Failure Mode [%]			
		Adhesive Failure at Dentin/Cement Interface	Adhesive Failure at Ceramic/Cement Interface	Cohesive Failure in Cement	Mixed Failure
IPS Empress CAD	RelyX U200	58.4	-	-	41.6
	Maxcem Elite	66.7	-	33.3	-
	Panavia SA	75	-	25	-
	Panavia V5	-	-	33.3	66.7
IPS e.max CAD	RelyX U200	75	-	8.3	16.7
	Maxcem Elite	83.3	-	-	16.7
	Panavia SA	75	-	25	-
	Panavia V5	58.4	-	-	41.6
IPS e.max ZirCAD	RelyX U200	50	-	25	25
	Maxcem Elite	66.7	16.6	-	16.6
	Panavia SA	50	25	-	25
	Panavia V5	58.4	41.6	-	-

**Table 3:** Statistical comparation of shear bond strength for thermocycled and non-thermocycled samples.

	Without Thermocycling		With Thermocycling		Bond Strength Decline		*t*-Test (df = 11)		Effect Size
Cement	M (MPa)	SD	M (MPa)	SD	M (MPa)	SD	*t*-Value	*p*-Value	Cohen’s *d_z_*
IPS e.max ZirCAD									
Panavia SA	18.31	1.357	2.55	0.558	15.77	1.424	38.36	<0.0001	11.07
Panavia V5	11.23	0.475	7.60	0.765	3.632	0.875	14.37	<0.0001	4.15
RelyX U200	22.73	1.148	1.95	0.520	20.78	1.196	60.18	<0.0001	17.37
Maxcem	13.29	1.185	2.32	0.182	10.97	1.212	31.35	<0.0001	9.05
IPS Empress CAD									
Panavia SA	16.94	1.533	2.98	0.667	13.96	1.924	25.13	<0.0001	7.25
Panavia V5	20.33	0.787	16.84	0.844	3.488	1.041	11.61	<0.0001	3.35
RelyX U200	15.13	1.953	3.36	0.260	11.77	1.895	21.51	<0.0001	6.21
Maxcem	15.48	1.158	3.37	0.229	12.11	1.274	32.92	<0.0001	9.50
IPS e.max CAD									
Panavia SA	9.888	0.926	3.209	0.588	6.679	1.162	19.91	<0.0001	5.75
Panavia V5	22.50	0.690	19.45	0.564	3.059	0.952	11.13	<0.0001	3.21
RelyX U200	8.228	0.696	3.135	0.595	5.092	0.908	19.42	<0.0001	5.61
Maxcem	8.428	0.867	2.058	0.445	6.370	0.739	29.87	<0.0001	8.62

**Table 4:** Ceramics and resin cement manufacturers.

Name	Type	Manufacturer
**Resin Cements**		
RelyX U200 A1	Self-adhesive, self-etch	3M ESPE (Maplewood, MN, USA)
Maxcem Elite A1	Self-adhesive, self-etch	Kerr (Brea, CA, USA)
Panavia SA Cement Universal A1	Self-adhesive, self-etch	Kuraray Noritake (Tokyo, Japan)
Panavia V5 A1	Adhesive	Kuraray Noritake (Tokyo, Japan)
**Ceramics**		
IPS Empress CAD HT A1	Leucite glass	Ivoclar Vivadent (Schaan, Liechtenstein)
IPS e.max CAD HT A1	Lithium disilicate	Ivoclar Vivadent (Schaan, Liechtenstein)
IPS e.max ZirCAD	Zirconia	Ivoclar Vivadent (Schaan, Liechtenstein)

## Data Availability

The data are available from the corresponding author upon reasonable request.

## Appendix A

### Appendix A.1. Validation of the Assumptions for Split-Plot ANOVA

The Mauchly’s test showed no deviations from sphericity (W = 0.99, χ^2^ = 0.46, *p* = 0.80). The Leven’s test indicated equal variances for IPS e.max CAD (F(3, 44) = 0.41, *p* = 0.75) and variance heterogeneity for IPS e.max ZirCAD (F(3, 44) = 6.13, *p* = 0.001) and IPS e.max CAD (F(3, 44) = 4.89, *p* = 0.005). Since in balanced designs (as in the current study) the F statistic is reasonably robust to variance heterogeneity we considered observed heterogeneity acceptable.

### Appendix A.2. Descriptive Statistics for the Examined Samples

**Table A1:** Descriptive statistics for the examined samples; N–number of samples, SD–standard deviation.

Cement	Ceramics	Shear Bond Strength [MPa]		N
		Mean	SD	
Panavia SA	IPS e.max ZirCAD	2.55	0.558	12
	IPS Empress CAD	2.98	0.667	12
	IPS e.max CAD	3.21	0.588	12
Panavia V5	IPS e.max ZirCAD	7.60	0.765	12
	IPS Empress CAD	16.84	0.844	12
	IPS e.max CAD	19.45	0.564	12
RelyX U200	IPS e.max ZirCAD	1.95	0.520	12
	IPS Empress CAD	3.36	0.260	12
	IPS e.max CAD	3.14	0.595	12
Maxcem	IPS e.max ZirCAD	2.32	0.182	12
	IPS Empress CAD	3.37	0.229	12
	IPS e.max CAD	2.06	0.445	12

### Appendix A.3. Results of the Post-Hoc Tukey’s HSD Test

**Table A2:** Tabularized representation of the results from the post-hoc Tukey’s HSD test for differences between means of the shear bond strength in particular samples defined by ceramics and cement type. The samples are sorted according to the means in ascending order. The means that are not significantly different from each other form a homogenous group and are marked by three black dots (“●●●”) in the same column. All means that do not share dots in the same column are significantly different from each other.

Cement	Ceramics	Shear Bond Strength [MPa]		Homogenous Groups						
		Mean	SE	1	2	3	4	5	6	7
RelyX U200	IPS e.max ZirCAD	1.95	0.158		●●●					
Maxcem	IPS e.max CAD	2.06	0.159		●●●					
Maxcem	IPS e.max ZirCAD	2.32	0.158		●●●		●●●			
Panavia SA	IPS e.max ZirCAD	2.55	0.158		●●●	●●●	●●●			
Panavia SA	IPS Empress CAD	2.98	0.163	●●●		●●●	●●●			
RelyX U200	IPS e.max CAD	3.14	0.159	●●●		●●●				
Panavia SA	IPS e.max CAD	3.21	0.159	●●●		●●●				
RelyX U200	IPS Empress CAD	3.36	0.163	●●●						
Maxcem	IPS Empress CAD	3.37	0.163	●●●						
Panavia V5	IPS e.max ZirCAD	7.60	0.158					●●●		
Panavia V5	IPS Empress CAD	16.84	0.163						●●●	
Panavia V5	IPS e.max CAD	19.45	0.159							●●●
